# Heads Up: Transcriptomics Reveal Functional Roles of Cannabis Glandular Trichome Stalks

**DOI:** 10.3390/plants15111624

**Published:** 2026-05-26

**Authors:** Paolo Miguel Siazon, Matthew Nolan, Qi Guo, Reilly Perovich, Tobias Kretzschmar, Jos C. Mieog

**Affiliations:** 1 Faculty of Science and Engineering, Southern Cross University, Lismore, NSW 2480, Australia; p.siazon.10@student.scu.edu.au (P.M.S.); qi.guo@scu.edu.au (Q.G.); reilly.perovich@ubc.ca (R.P.); tobias.kretzschmar@scu.edu.au (T.K.); 2 Commonwealth Scientific and Industrial Research Organisation, St. Lucia, Brisbane, QLD 4067, Australia; matt.nolan@csiro.au; 3 Faculty of Science, The University of British Columbia, Vancouver, BC V6T 1Z4, Canada

**Keywords:** *Cannabis sativa*, glandular trichome, transcriptomics, transporter, plasmodesmata, source-sink

## Abstract

*Cannabis sativa* glandular trichomes (CsGT) are the primary sites of cannabinoid biosynthesis. They consist of a metabolically active head and a structurally supportive stalk. While the biosynthetic role of the head is well established, understanding of the functional contribution of the stalk has lagged. Here, we performed an integrated proteomic and transcriptomic analysis of isolated CsGT head and stalk tissues to identify their distinct roles. While proteomic analysis found 35 proteins higher in the stalk compared to the head, the transcriptomic analyses suggest the true functionality of the stalk, with 6018 genes showing higher expression in this tissue. Marker gene analysis showed a predominantly epidermal signature for head cells, while stalk cells also showed signatures for vascular cell types. Congruently, the transcriptomic results showed genes with higher expression in the head associated with specialized secondary metabolite production and accumulation, while the stalk displayed enrichment for genes related to active growth, vascular, apoplastic and symplastic transport, as well as sugar and hormone signalling. These findings support a model in which the stalk is a complex epidermal tissue, highly distinct from the head, that facilitates metabolite transport, signalling, and precursor supply to the glandular head. This study provides the first multi-omics characterization of CsGT stalk function, identifying it as a key contributor to trichome productivity and a potential target for optimizing cannabinoid yield.

## 1. Introduction

**Figure 1:**
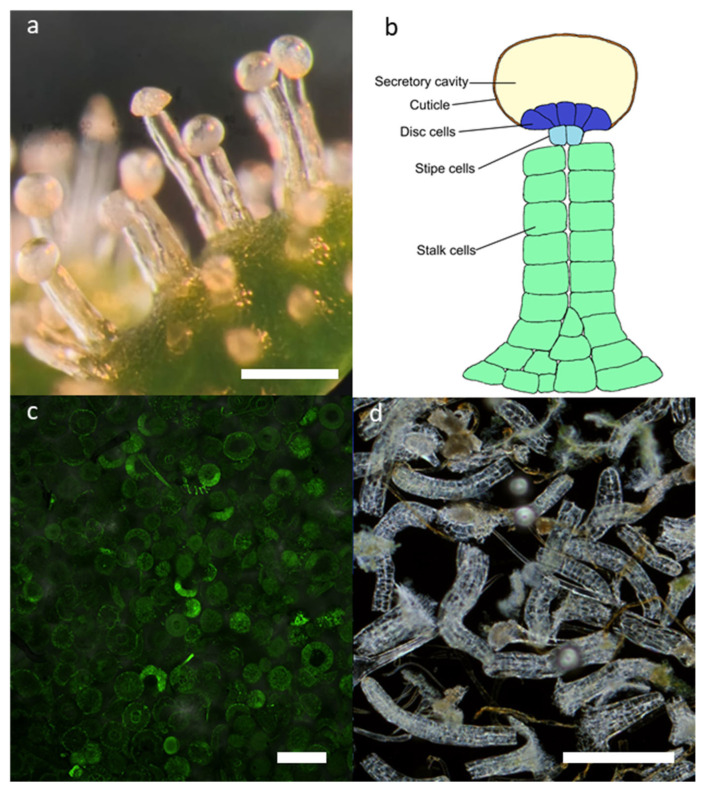
Morphology of *C. sativa* glandular trichomes (CsGT) Hindu Kush cultivar at week 6 of flower development. (**a**) Microscopy image of intact CsGTs on leaf tissue showing the stalk and secretory head; (**b**) schematic diagram of a CsGT structure; (**c**) confocal laser microscopy image of CsGT disc cells after isolation showing autofluorescence; (**d**) isolated stalk cells from CsGTs using darkfield microscopy. White scale bars represent 100 µm.

*Cannabis sativa* produces a wide range of specialized metabolites, most notably cannabinoids and terpenes, which underpin its medicinal value [1,2,3]. These are synthesized and stored in the *C. sativa* glandular trichomes (CsGTs); specialized epidermal structures predominantly present on floral tissues of the female plant (Figure 1a) [4]. CsGTs are composed of a ‘head’ and a ‘stalk’ (Figure 1b). The head is formed by secretory disc cells, which synthesize cannabinoids and terpenes that accumulate in the subcuticular headspace apical to the disc cells [5], called the storage cavity. The head is connected to the stalk via a layer of stipe cells (Figure 1b) [5,6,7]. The stipe cell layer forms the weakest link in the trichome, allowing the heads to be relatively easily isolated from the rest of the plant via shearing, a feature that is taken advantage of during mechanical separation via tumbling or sieving plant biomass when producing for instance hashish [8]. While the head has a well-defined structure that only varies to some extent in size and the number of secretory cells within the disc cell ring [9], the stalk is a complex structure that consists of six peripheral cells surrounding a central core and shows large variability in size across development within an individual plant/variety and across accessions [5]. Head size and disc cell number have been positively associated with cannabinoid content [9], and similar has been suggested for stalk length [5]. However, the functional role of the stalk remains poorly characterized and has received substantially less molecular investigation than the glandular head.

CsGT development proceeds through three defined stages: pre-secretory, secretory, and post-secretory [2]. Histological, developmental, and molecular studies describe the pre-secretory phase as a period of rapid cell division, early cell fate specification, and plastid differentiation with little or no secondary metabolite accumulation. This is followed by a secretory phase marked by maximal gland expansion, high metabolic flux, and rapid accumulation of cannabinoids and terpenes, before transitioning into a post-secretory phase associated with declining biosynthetic activity and progressive cellular senescence [5,6]. Proteomic profiling on isolated CsGT heads has supported this, revealing coordinated shifts in plastid-associated proteins, central carbon metabolism, and secretory machinery that parallel trichome maturation and precede peak secondary metabolite accumulation [2].

Cannabinoids are C21-C22 terpenophenolic compounds that originate from the condensation of a polyketide precursor, olivetolic acid, with the isoprenoid precursor geranyl diphosphate, with subsequent steps yielding the major acids Δ9-tetrahydrocannabinolic acid (THCA) and cannabidiolic acid (CBDA), as well as many minor cannabinoids [10]. Monoterpenes, derived via the plastidial methylerythritol phosphate (MEP) and cytosolic mevalonate pathways, contribute to the distinctive aroma of cannabis and serve roles in plant-environment interactions, including defence [11]. Cannabinoids and terpenes are synthesized by the CsGT disc cells at the base of the glandular head and are stored into a subcuticular secretory cavity formed between the disc cells and the overlying cuticle, where these amphiphilic metabolites accumulate at high concentration [2]. Elite cultivars selected for medicinal and recreational use routinely exhibit extremely high cannabinoid accumulation in floral tissues, with total cannabinoid content approaching ~30% of inflorescence dry weight, reflecting the remarkable biosynthetic capacity and strong sink function of CsGTs [12]. This extreme metabolic output imposes substantial demands on precursors, reducing agents, and ATP, thereby requiring a substantial influx of photoassimilates and tight coordination between primary and secondary metabolism.

Given their non-photosynthetic nature and extreme biosynthetic activity, CsGT heads function as strong sink tissues that require the continuous import of photoassimilates from surrounding flower tissues to sustain high levels of secondary metabolism [2,9,13,14,15]. Consequently, CsGTs must be equipped with specialized molecular machinery to support carbon import, energy provision, and sustained biosynthetic activity. In addition to their metabolic role, CsGTs are integrated into whole plant communication networks and have been implicated in the translocation of hormones, small RNAs and proteins, as well as in plant defence responses [14,16,17]. The position of the stalk between the metabolically active head and the photosynthetically active leaf, which also supplies the connection to the rest of the plant body, strongly suggests a role in mediating exchange of metabolites, nutrients, and signals [18,19]. Recent findings from *C. sativa* suggest that the stalks supply sugar to the head [13,15].

Research on CsGT trichomes has almost exclusively been focused on the heads [2,6,14,20] largely due to the technical difficulty of isolating pure stalk fractions in sufficient quantities to facilitate omics analyses. Only one study to date has investigated the stalks specifically using an Information Dependent Acquisition (IDA) proteomics approach [13]. The results suggest that CsGT stalks are involved in light-dependent photosynthesis, and are proposed to deliver sugars and reducing equivalents from surrounding photosynthetic tissues to the head in support of cannabinoid biosynthesis [13]. However, due to the limited number of identified proteins and possible contamination from other tissues, details on the stalk’s role in sugar transport and secondary metabolite biosynthesis remained unclear.

To address this knowledge gap, we developed a method to obtain cleanly separated fractions of CsGT heads and stalks, enabling independent molecular analysis of each tissue. Both quantitative proteomics and transcriptomics were employed to investigate functional differentiation between the two tissues. This multi-omics investigation on glandular trichome specialization provides the first comprehensive, tissue-level resolution characterization of CsGTs, offering new insights into trichome biology.

## 2. Results

### 2.1. Isolation of Cannabis Glandular Trichome Heads and Stalks

CsGT heads and stalks were isolated using 25 µm and 75 µm sieves, respectively, resulting in clearly distinct fractions (Figure 1c,d). Isolated heads appeared as compact, oblate spheroid structures (Figure 1c), whereas stalks were cylindrical and elongated (Figure 1d). Microscopy-based counting confirmed a high purity of the isolated fractions, with 99.8% heads and 90.9% stalks, respectively (Figure S1).

### 2.2. High-Level Description of Proteomic and Transcriptomic Results

**Figure 2:**
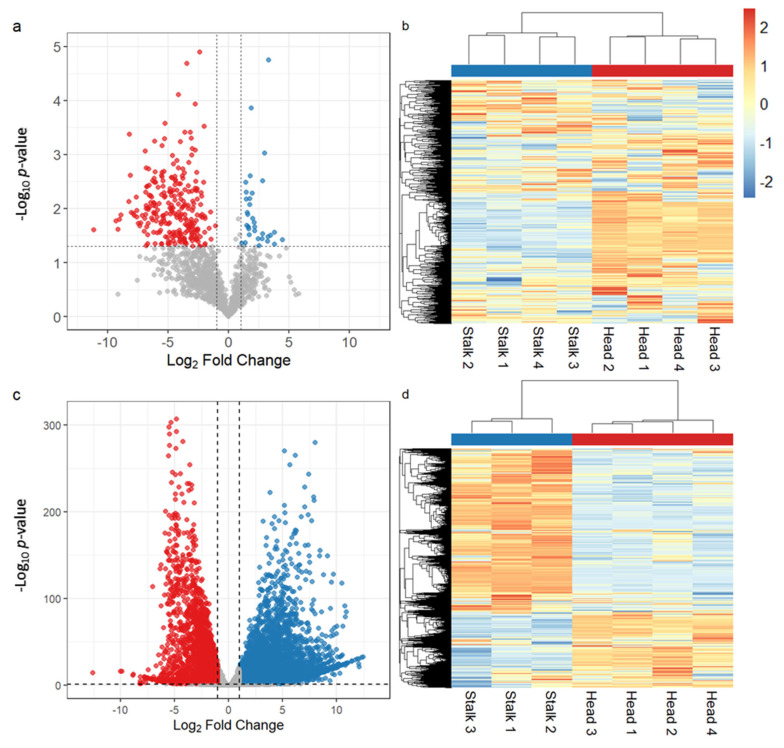
Comparative analysis of CsGT stalk and head cells from the Hindu Kush cultivar at week 6 of flower development. Panels show the proteome (**a**,**b**) and transcriptome (**c**,**d**) comparisons with volcano plots (**a**,**c**) and cluster diagrams (**b**,**d**). Volcano plots depicting differential abundance of proteins (**a**) or differentially expressed genes (**c**) between stalks and heads. Proteins/genes with significantly higher abundance or expression in stalk or head tissues are coloured blue or red, respectively, while non-significant changes are grey. Thresholds were log_2_fold−change ≥ 1 and *p*.adj ≤ 0.05. Dashed vertical lines indicate the log_2_fold−change thresholds, while the dashed horizontal line indicates the *p*.adj significance threshold. Hierarchical clustering heatmaps are shown for proteome (**b**) and transcriptome (**d**) data. Data were row-scaled (z−score) to highlight relative abundance patterns between stalk and head tissue. Sample sizes were *n* = 4 for the proteome, and *n* = 4 (Head) and *n* = 3 (Stalk) for the transcriptome.

In total, 399,488,302 paired-end reads were generated across eight samples from both CsGT tissues, averaging 57,069,757 reads per sample (Table S1). SWATH-MS analysis resulted in the identification of 1285 unique proteins, while RNA-seq analysis detected 22,576 unique transcripts across both tissues. A cut-off of log_2_fold change ≥ |1| and a false discover rate-adjusted *p*-value of 0.05 were used to define the differentially abundant proteins (DAPs) and differentially expressed genes (DEGs). In the proteome, 300 proteins (23.3%) were differentially abundant, with 35 proteins (2.7%) enriched in CsGT stalks, while 265 proteins (20.6%) were more abundant in heads (Table S2). At the transcript level, 2442 transcripts were uniquely detected in stalks, while 1140 transcripts were uniquely detected in heads. A total of 9330 transcripts (41.3%) were strongly differentially expressed, including 6018 higher in stalks (26.7%) and 3312 higher in heads (14.7%) (Table S3). Volcano plots highlighted broad and coordinated differences in abundance and expression between tissues (Figure 2b,d).

Principal component analysis (PCA) (Figure S2) of both proteomic and transcriptomic datasets revealed clear separation between stalk and head samples. For the proteome data, PC1 accounted for 40.0% of the total variance and separated the two tissue types, while the transcriptomic PC1 accounted for 98.0% of the variance (Figure S2). Hierarchical clustering and volcano plots (Figure 2a,c) supported PCA results, demonstrating that stalk and head tissues possess distinct molecular identities at both RNA and protein levels.

Directional concordance between identified proteins and their respective RNA was evaluated for the DAPs (Table S4). Among the DAPs, 165 proteins (61.9%) showed concordant regulation with their corresponding mRNA levels. Within the concordant subset, 15 proteins were higher in the stalk at both protein and mRNA levels (Up/Up, 5.64%), whereas 150 proteins were lower in the stalk at both levels (Down/Down, 56.4%). Discordant regulation was observed for 101 proteins (37.97%), including 22 proteins higher at the protein level but lower at the RNA level (Up/Down, 8.27%), and 79 proteins lower at the protein level but higher at the RNA level (Down/Up, 29.7%).

### 2.3. Comparison of Most Abundant Transcripts and Proteins

Analysis of the proteins with the highest relative abundance and most abundant transcripts further confirmed functional divergence. In CsGT stalks, these were primarily associated with RNA processing, protein synthesis, and intracellular trafficking (Table S5). Proteins involved in vesicle-mediated transport and secretion were highly abundant, including ER–Golgi and intra-Golgi vesicle trafficking. Stalk-abundant features further included chloroplast-localized proteins related to photosynthesis and lipid metabolism. Abundant transcripts further included hormone- and stress-responsive genes, cell wall–associated genes, and ubiquitin-related factors. In contrast, the most abundant transcripts and proteins in CsGT heads included enzymes involved in redox-related processes and energy metabolism (Table S5). Highly abundant features also included enzymes associated with fatty acid and isoprenoid as well as cannabinoid biosynthesis.

### 2.4. Marker Gene-Based Inference of Stalk Molecular Signature

**Figure 3:**
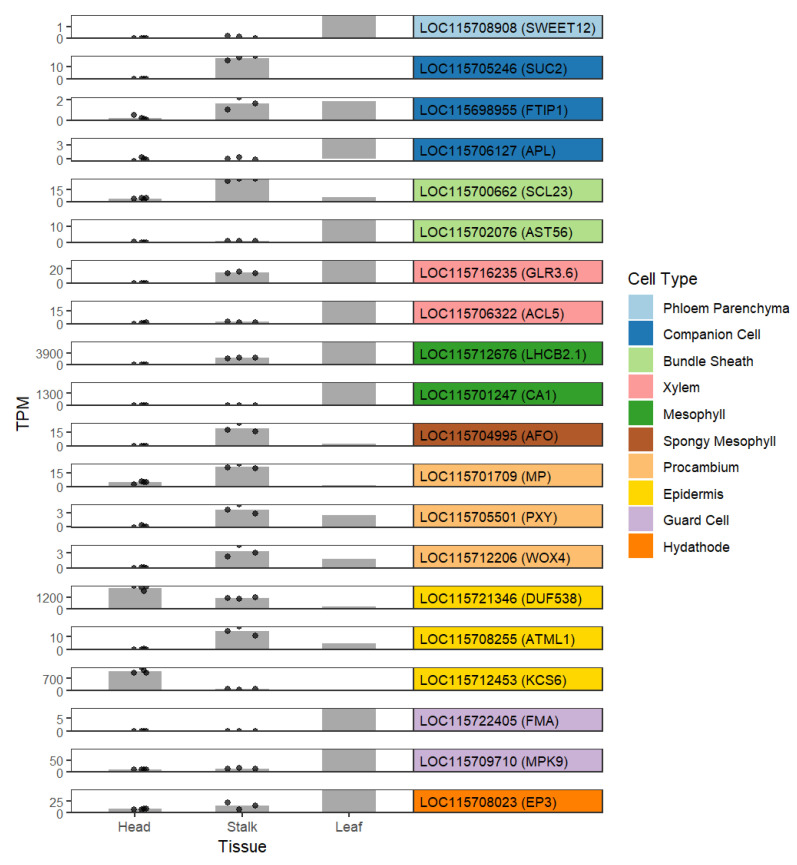
Expression of marker gene homologs in head (*n* = 4), stalk (*n* = 3) and leaf tissues, with expression levels reported in transcripts per million (TPM). Grey bars show mean expression per tissue, with individual points representing biological replicates. Data for heads and stalks were generated in this study, while leaf expression mean values were obtained from the Cannabis Expression Atlas [22] Gene names are indicated on the facet strips on the right, and strip colours correspond to the annotated cell type of each marker. Abbreviations: *SWEET12*—*Sugar Will Eventually be Exported Transporter 12*; *SUC2*—*Sucrose transport protein 2*; *FTIP1*—*Flowering locus T interacting protein 1*; *APL*—*Altered phloem development*; *SCL23*—*Scarecrow-like 23*; *AST56*—*Arabidopsis sulfate transporter 56*; *GLR3.6*—*Glutamate receptor-like 3.6*; *ACL5*—*Acaulis 5*; *LHCB2.1*—*Light-harvesting chlorophyll a/b-binding protein 2.1*; *CA1*—*Carbonic anhydrase 1*; *AFO*—*Abnormal floral organs*; *MP*—*Monopteros*; *PXY*—*Phloem intercalated with xylem*; *WOX4*—*Wuschel-related homeobox 4*; *DUF538*—*Domain of unknown function 538*; *ATML1*—*Arabidopsis thaliana meristem layer 1*; *KCS6*—*3-Ketoacyl-CoA synthase 6*; *FMA*—*Fama*; *MPK9*—*Mitogen-activated protein kinase 9*; *EP3*—*Endochitinase EP3*.

To assess whether CsGT stalk and head transcriptomes reflected distinct cellular identities, we used cell-type-specific marker transcripts previously defined in Arabidopsis leaf single-cell RNA-seq [21] (Table S6, Figure 3). Congruent with trichomes being epidermal outgrowths, all three epidermis markers showed higher expression in both CsGT tissues compared to the young leaf. *Domain of unknown function 538* (*DUF538*) and *3-Ketoacyl-CoA synthase 6* (*KCS6*) were enriched in heads, while the homologue of *Arabidopsis thaliana meristem layer 1* (*ATML1*) marker was higher in stalk.

Head samples showed limited expression of non-epidermal marker genes (Figure 3). Contrastingly stalks expressed several marker genes associated with companion cells (*Sucrose transport protein 2* (*SUC2*) and *Flowering locus T interacting protein 1* (*FTIP1*)), bundle sheath (*Scarecrow-like 23* (*SCL23*)), xylem (*Glutamate receptor-like 3.6* (*GLR3.6*)), spongy mesophyll (*Abnormal floral organs* (*AFO*)) and procambium (*Monopteros* (*MP*), *Phloem intercalated with xylem* (*PXY*), and *Wuschel-related homeobox 4* (*WOX4*)) at levels that were comparable or exceeded expression in young leaves. Together, these patterns suggest that CsGT head cellular identity mainly reflects their epidermal origin, while stalk cells exhibit a more differentiated identity that was associated with (pre-)vascular components in addition to epidermis.

### 2.5. KEGG Pathway Analyses

**Figure 4:**
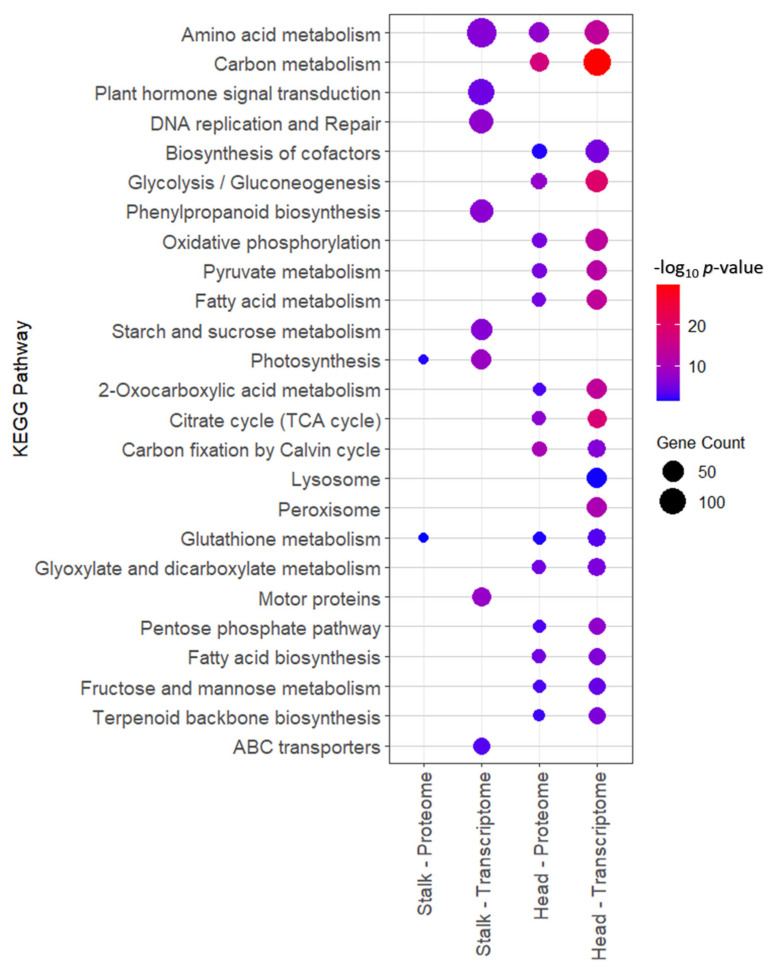
KEGG pathway comparison analysis of DAPs and DEGs between CsGT heads and stalks isolated from the flower of the *C. sativa* cultivar Hindu Kush at week 6 of flower development. Dot size represents the number of DAPs (proteome) or DEGs (transcriptome) mapped to each pathway. Dot colour indicates the *p*−value, with a threshold set at *p*.adj < 0.05 to denote statistical significance.

KEGG pathway enrichment analyses revealed pronounced functional divergence between CsGT stalk and head tissues at both transcriptomic and proteomic levels (Figure 4). In stalks, the proteome showed significant enrichment for “Photosynthesis” and “Glutathione metabolism”. Congruently, “Photosynthesis” was enriched in the stalk transcriptome, which was additionally enriched for “Amino acid metabolism”, “Plant hormone signal transduction”, “DNA Replication and repair”, “Phenylpropanoid biosynthesis”, “Starch and sucrose metabolism”, “Motor proteins”, and “ABC transporters”. For plant hormone signal transduction, genes associated with auxin, cytokinin, gibberellin, abscisic acid, brassinosteroid, jasmonic acid, and salicylic acid-related pathways were enriched in the stalk (Figure S3).

In contrast, the CsGT head proteome demonstrated enrichment for central carbon and energy metabolism, including “Amino acid metabolism”, “Carbon metabolism”, “Biosynthesis of cofactors”, “Glycolysis/Gluconeogenesis”, “Oxidative phosphorylation”, “Pyruvate metabolism”, “Fatty acid metabolism”, “2-oxocarboxylic acid metabolism”, “Citrate cycle (TCA cycle), “Carbon fixation by Calvin cycle, “Glutathione metabolism”, “Glyoxylate and dicarboxylate metabolism”. KEGG enrichment of the head transcriptome identified the same core metabolic pathways, and further showed enrichment for “Lysosome” and “Peroxisome”.

Gene Ontology (GO) biological process (BP) enrichment analyses showed that genes and proteins associated with cell cycle regulation and division, cytoskeletal support, microtubule-based movement, nitrogen and nucleotide synthesis, photosynthesis, and protein phosphorylation were enriched in the stalk (Figure S4).

In contrast, genes and proteins associated with ubiquitin-dependent processes, respiration and vesicular trafficking through localization were enriched in the head (Figure S3). The head is also enriched for processes related to translational elongation and protein turnover.

### 2.6. Cannabinoid and Terpenoid Biosynthesis Expression Patterns

**Figure 5:**
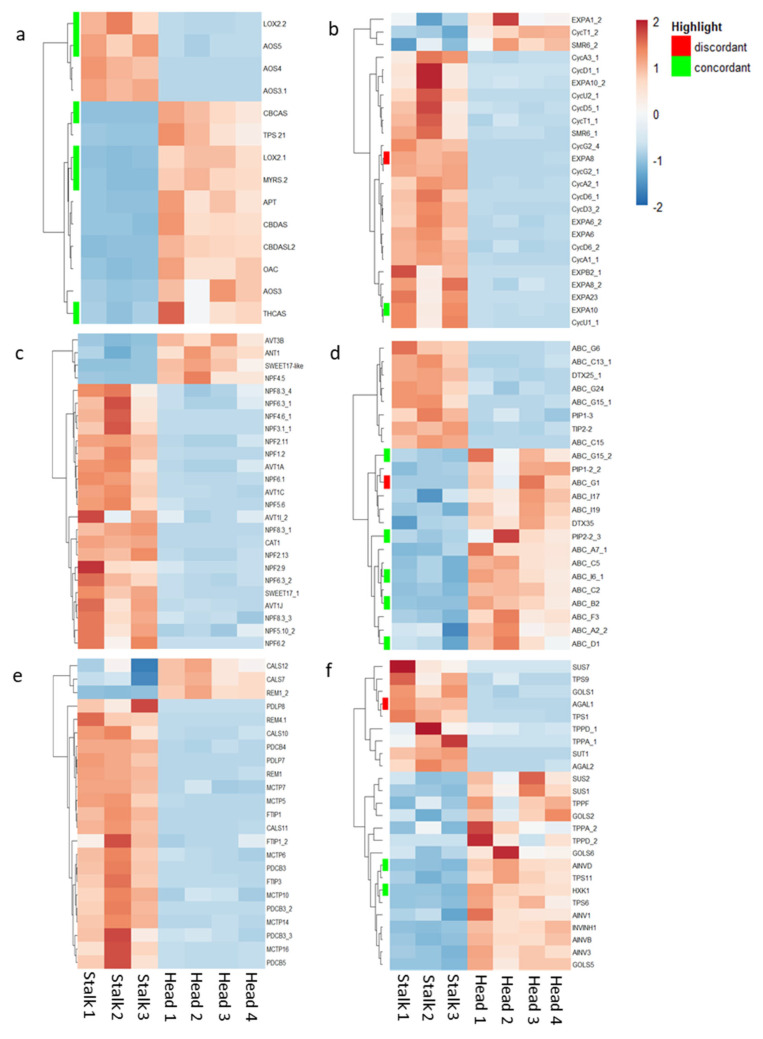
Heatmaps showing the expression patterns of selected transcripts in CsGT stalks and heads isolated from the inflorescence of Hindu Kush at week 6 of flower development. Panels represent gene groups related to (**a**) secondary metabolite synthesis genes, (**b**) growth-related genes, (**c**) vascular transport-related genes, (**d**) membrane transport-related genes, (**e**) plasmodesmata-related genes, and (**f**) source-sink related genes. Values are based on normalized transcript counts and are presented as row-scaled (z−score) expression profiles. Statistical significance of expression of treatment group comparisons at *p*.adj < 0.05 and log_2_fold−change > 1. Annotation bars indicate detection at the proteome, with green indicating concordant trends between protein and transcript abundance and red indicating discordant trends.

Transcripts and proteins involved in secondary metabolite synthesis show distinct expression patterns (Figure 5a, Table S7) between tissues, most of them higher in heads.

*Allene oxide synthases* (*AOS*) and *Lipoxygenase* (*LOX*), which are associated with the early steps of fatty acid metabolism, showed higher transcript abundance in stalk tissue. In contrast, transcripts associated with cannabinoid and terpene biosynthesis, such as *Tetrahydrocannabinolic acid synthase* (*THCAS*), *Cannabichromenic acid synthase* (*CBCAS*), *Cannabidiolic acid synthase* (*CBDAS*), *Olivetolic acid cyclase* (*OAC*), *2-acylphloroglucinol 4-prenyltransferase* (*APT*), *Limonene synthase 21* (*TPS21*), *Myrcene synthase* (*MYRS*), and *LOX2_1* showed higher expression in the head. 6 of these transcripts (*AOS5*, *LOX2*, *THCAS*, *CBCAS*, *MYRS*, *LOX2_1*) were also detected at the proteomic level, and all 6 were directionally concordant with their respective transcripts.

### 2.7. Growth-Related Expression Patterns

Cyclins and cyclin-dependent kinases regulate cell cycle progression for cell division [23], while expansins mediate cell wall loosening to enable cell expansion [24]. Differential expression was observed among *Cyclins* (*CYC*), *Cyclin-dependent Kinases* (*CDK*s), *CDK inhibitors* (*CKI*s), and α- and β-Expansins (*EXPA/EXPB*) between trichome stalk and head tissues. A total of 54 genes were differentially expressed, comprising 36 cyclin-related genes (21 *CYCs*, 9 *CDK*s, and 6 *CKI*s) and 18 expansins. The majority of these genes were higher in stalks, with 47 transcripts more highly expressed in the stalk and 7 in the head.

The expression patterns of the top 25 genes ranked by variance are shown in Figure 5b (with full expression data in Table S8). Transcripts with higher expression in the head included one cyclin (*CycT1_2*), one CKIs (*SMR6_2*) and one expansin (*EXPA1_2*). Transcripts with higher expression in the stalk included 13 cyclins members of the D, A, U, T and G families, one CKI (*SMR6_1*), and eight expansins. Of the differentially expressed genes, *EXPA10* showed concordant RNA and protein upregulation in stalks, whereas *EXPA8* was discordant, with higher RNA expression in stalks but protein more abundant in heads.

### 2.8. Expression Related to Vascular Transport

#### 2.8.1. Amino Acid, Sugar, and Peptide Transporters

Differential expression was observed among *amino acid vacuolar transporters* (*AVT*) and *cationic amino acid transporters* (*CAT*), *SWEET* transporters, and *Nitrate transporter1/Peptide transporter family transporters* (*NPF*), with the majority more highly expressed in the stalk. A total of 47 genes, with 12 amino acid transporters, 7 SWEET transporters and 28 NPF transporters were differentially expressed. Of these, 8, 5, and 22 transcripts, respectively, showed higher expression in stalks, while 4, 2, and 6 transcripts showed higher expression in heads. The expression patterns of the top 25 transcripts ranked by variance are shown in Figure 5c, with full expression data in Table S9. No corresponding proteins were detected for these transporters in the proteomic dataset.

Transcripts with higher expression in the head included two amino acid transporters (*AVT3B, ANT1*), one SWEET transporter (*SWEET17-like*), and one peptide transporter (NPF4.5). Transcripts with higher expression in the stalk included 15 peptide transporters, five amino acid transporters, and one SWEET transporter (*SWEET 17*).

#### 2.8.2. ABC, DTX Transporters and Aquaporins

*ATP-binding cassette (ABC) transporters*, *Multidrug and toxic compound extrusion transporters*, also referred to as *Detoxification* (*MATE/DTX*), and aquaporins were found to be differentially expressed between trichome stalk and head tissues. In total, 48 *ABC* transporter transcripts (26 up in stalks, 22 up in heads), 20 *DTX* transporter transcripts (15 up in stalks, 5 up in heads), and 17 aquaporin transcripts (12 up in stalks, 5 up in heads) were differentially expressed. However, in contrast to symplastic transporters, the split between stalk- and head-expressed transcripts was more balanced for ABC transporters. The expression patterns of the top 25 transcripts ranked by variance are shown in Figure 5d, with full lists provided in Table S10.

Transcripts with higher expression in the head included five ABC transporters of the ABCG and ABCC subfamilies, two aquaporins (*PIP-3*, *TIP2-2*), and one DTX (*DTX25_1*). In stalks, 17 transcripts showed higher expression, including 12 ABC transporters of the ABCA, ABCB, ABCC, ABCD, ABCF, ABCG, and ABCI subfamilies, two aquaporins (*PIP1-2_2*, *PIP2-2_3*), and one DTX (*DTX35*).

Of the ABC transporters, *ABCD1*, *ABCI6_1*, and *ABCB2* were also detected at the protein level and showed concordant RNA/protein expression, whereas *ABCG1* showed discordant expression, with up-regulation in the head at the RNA level but higher protein abundance in the stalk. Among aquaporins, *PIP2-2_3* was also detected at the protein level and showed concordant expression. No corresponding proteins were detected for the DTX transcripts.

#### 2.8.3. Plasmodesmata

DEGs were prominently associated with plasmodesmata (PD) structure and regulation between CsGT stalks and heads. These genes included members of several PD-associated families, such as *Plasmodesmata-located proteins* (*PDLPs*), *Plasmodesmata Callose-binding proteins* (*PDCB*), *Callose synthases* (*CALS*), *Remorin proteins* (*REM*), *Flowering locus T—interacting proteins* (*FTIP*), and *Multiple C2 domains and transmembrane region proteins* (*MCTP*). In total, 23 PD-related transcripts were differentially expressed, of which 20 showed higher expression in stalks and 3 showed higher expression in heads. Expression patterns of the transcripts are shown in Figure 5e, with full gene lists provided in Table S11.

Transcripts with higher expression in the head included two callose synthases (*CALS12*, *CALS7*) and one remorin (*REM1_2*). Transcripts with higher expression in the stalk included six MCTP genes, five PDCBs, three FTIPs, two PDLPs (*PDLP8*, *PDLP7*), two remorins (*REM4.1*, *REM1*), and two CALS (*CALS10*, *CALS11*). No corresponding PD-associated proteins were detected in the proteome dataset.

### 2.9. Expression Patterns Linked to Source-Sink Dynamics

Several DEGs were associated with source–sink metabolism, sugar signalling, raffinose-family oligosaccharide metabolism, and sugar transport. In total, 27 transcripts were differentially expressed, with 9 showing higher expression in the stalk and 18 showing higher expression in the head. Expression patterns of the top transcripts ranked by variance are shown in Figure 5f, with full gene lists provided in Table S12.

Transcripts with higher expression in the stalk included *Sucrose transport protein* (*SUT1*), and *Sucrose synthase* 7 (*SUS7*), along with two *α-Galactosidases* (*AGAL1*, *AGAL2*), two *Trehalose phosphate synthases* (*TPS1*, *TPS9*), two *Trehalose phosphate phosphatases* (*TPPA_1*, *TPPD_1*) and one *Galactinol synthase* (*GOLS1*).

Transcripts with higher expression in the head included four *Alkaline/Neutral Invertases* (*AINV*), three GOLS, two SUS (*SUS1*, *SUS2*), two TPS (*TPS6*, *TPS11*), two TPPs (*TPPA_2*, *TPPD_2*), and one *Invertase inhibitor* (*INH1*). Among these genes, *AGAL1*, *HXK1*, and *AINVD* were also detected at the protein level. *HXK1* and *AINVD* showed concordant RNA and protein expression, whereas *AGAL1* showed discordant expression, with higher RNA levels in stalks but higher protein abundance in heads.

## 3. Discussion

### 3.1. Successful Separation of Stalk and Head Tissues Allows Powerful Comparative Multi-Omics

Trichome productivity is the main driver of Cannabis psychoactive potency and has been strongly shaped by artificial selection. CsGT heads of modern medicinal cultivars are larger and contain more disc cells than those of their traditional landraces [9]. Stalks are also longer and larger in high-cannabinoid cultivars [5], suggesting a link between stalk development and cannabinoid biosynthetic capacity.

To date, isolated trichome stalks have not been studied in other species; thus, it is unknown whether the functional complexity found for CsGTs is a common feature of glandular trichomes. However, other species often possess relatively simple stalk structures composed of only a few cells [25,26,27,28], raising the possibility that the enhanced transport- and vascular/pre-vascular-associated signatures observed here may be more pronounced in, or even limited to, more complex stalk structures such as found on *Cannabis*. Further comparative studies across glandular trichome systems will be required to determine this.

Despite these positive associations, the functional contribution of CsGT stalks remains poorly understood. To date, only one study has directly investigated stalk function, reporting enrichment of proteins linked to energy production, photosynthesis, and detoxification [13] though interpretation was limited by head tissue contamination and low proteomic resolution.

Here, an optimized isolation workflow enabled reproducible isolation of highly enriched stalk and head fractions. Microscopy confirmed the purity of tissues after isolation, indicating successful separation (Figure 1c,d). Separation efficiency was very high with only 2.4% of heads in the stalk fraction (Figure S1), indicating that contamination was highly unlikely to be a driver of the low number of differentially abundant proteins. Cystolith formation is thought to be linked to early leaf ontogeny [29], which is consistent with the contamination in our samples appearing as mature cystoliths. These primarily act as calcium reservoirs [30] and can be expected to have a relatively low gene expression. Therefore, we assume that the cystolith contamination seen in the stalk samples had a negligible impact on our transcriptomics dataset. Instead, the limited number of stalk-enriched proteins likely reflects the dominance of shared core cellular and housekeeping proteins between the two tissues, both derived from epithelial cells, combined with the inherent bias of mass spectrometry toward detecting higher-abundance proteins. PCA and hierarchical clustering of both datasets cleanly separated stalk and head samples, supporting successful separation and highlighting their distinct molecular identities (Figure 2 and Figure S2). While the directional concordance between transcript and protein was moderate (Table S4), it was consistent with previous plant multi-omics studies [31,32,33], likely reflecting post-transcriptional regulation, protein turnover, and lower detection sensitivity in proteomic analyses [34]. This can also explain why this and previous studies [13] found only limited evidence for a functional role of the stalk based on the proteome alone (Figure 2). The proteome-transcriptome discordance likely reflects both biological decoupling and methodological biases in protein versus RNA quantification. Post-transcriptional regulation, differences in translation efficiency, transcript turnover, and protein stability [35] are all known to be able to lower the correspondence between DEGs and DAPs. From a technical perspective, mass spectrometry-based proteomics is inherently limited by lower sensitivity compared to RNA-seq, incomplete proteome coverage, and variability in protein extraction and detectability [36].

Further supporting good fractionation, our study confirmed that disc cells are specialized cells dedicated to cannabinoid and terpene biosynthesis, with terpene backbone biosynthesis (Figure 4) and cannabinoid biosynthetic transcripts and proteins OAC, THCAS, CBDAS, and CBCAS highly overabundant in the head (Figure 5a). Intriguingly, we found that AOS expression was higher in the stalk, together with the protein ATP-citrate synthase, a key enzyme generating precursors for fatty acid elongation [37], and *nsLTP1* transcripts, involved in lipid transport [38] (Table S5), suggesting that these genes may be associated with early steps in fatty acid metabolism in the stalk, potentially contributing to precursor supply for downstream processes in the head. Additionally, key DEGs, including *AOS*, *LOX2*, *LOX2_1*, *EXPA10*, *ABCD1*, *ABCI6_1*, *ABCB2*, *HXK1*, and *AINVD* (Figure 5a,b,d,f) showed concordance with corresponding DAPs. These results demonstrate the generation of robust datasets suitable for biological interpretation.

### 3.2. The Stalks Exhibit a Complex Cellular Profile While the Heads Are True-to-Origin and Highly Specialised

Recent advances in single-cell RNA (scRNA) sequencing have demonstrated that plant cell types can be identified through characteristic transcriptional signatures. In Arabidopsis, scRNA studies of leaf tissues defined distinct marker genes for specific cell types, including vascular, mesophyll, and epidermal cell types [21]. Although typically applied to single-cell datasets, conserved cell-type marker genes can also help infer cellular composition in bulk tissue samples when physical separation of individual cell types is not feasible [39]. Because core cellular processes are highly conserved across angiosperms, Arabidopsis-derived marker sets offer a useful reference for comparative analyses in non-model species.

Marker gene analysis shows that the stalk expresses transcriptional signatures associated with vascular-related cell types (Figure 3), particularly phloem companion cells and procambium. Companion cells are central to phloem loading and long-distance transport of sugars, metabolites, and signalling molecules [40], while the procambial-associated markers are linked to vascular differentiation and patterning [41]. CsGTs form from epidermal cells, initially as sessile structures with the secretory head positioned directly on the epidermis [1]. During flower maturation, trichomes undergo further morphological and metabolic changes, resulting in the mature secretory phase trichomes consisting of several distinct cell types, including stalk, stipe, and secretory disc cells [2,42,43]. Marker gene signatures suggest that epidermally derived stalk cells adopt additional transcriptional programs beyond epidermal functions during specialisation, suggesting potential roles in transport of water, nutrients, and metabolites towards the head. Recent microscopy studies on CsGT morphology revealed a hollow core in the stalk surrounded by six cells, structurally similar to phloem tissue [5], further supporting the similarity between the stalk and phloem. Notably, the epidermal marker *ATML1* showed higher expression in the stalk, indicating that at least some stalk cells retain core epidermal identity. Transcripts of phloem-associated transcription factors and development genes were also enriched in the stalk (Figure S5), further supporting vascular-related regulatory signatures. These findings raise the possibility that CsGT stalk cells may partially engage vascular-associated developmental programs during differentiation. This raises the question of whether the stalk represents a case of partial adoption of vascular developmental programs or whether these features instead reflect evolutionary convergence between transport-supporting and secretory tissues, as suggested in other specialised secretory systems such as laticifers [44] and oil glands [45].

In contrast, the head shows strong epidermal specialization consistent with cuticle formation and lipid biosynthesis functions. DUF538 proteins contribute to cuticle formation and epidermal differentiation [46], while KCS6 catalyzes the elongation of very-long-chain fatty acids, a critical step in cuticular wax and cutin biosynthesis [47], likely supporting the formation of the cuticle layer that surrounds the secretory cavity.

### 3.3. The Stalk Gene Expression Supports Active Growth, Structural Support and Hormonal Signalling

KEGG pathway analysis revealed DNA replication and repair, motor proteins, and phenylpropanoid biosynthesis (Figure 4) to be highly enriched in stalks even six weeks after floral initiation, which has been identified as the latter part of the secretory phase [2]. Further transcript level analysis revealed increased expression of cyclins, CDK inhibitors, and expansins (Figure 5a and Table S8) which are genes linked to active cell division [48], cell wall remodelling and structural strengthening [49]. The presence of motor proteins is potentially also a mechanism by which the stalk provides structural support to the head, likely through mediating the transport and deposition of cell wall materials. This supports a model in which coordinated cell division and cell wall loosening contribute to continued stalk elongation well into the secretory phase [24,50]. Furthermore, the enrichment of the phenylpropanoid biosynthesis pathway in the stalks (Figure 4) suggests that lignin and lignin-like compounds derived from phenylpropanoid monolignols [51] are made available to strengthen primary cell walls and impart mechanical strength during growth, reinforcing wall stiffness and structural integrity as seen for vascular tissues [49,52]. This increases mechanical strength and potentially helps to stabilize the CsGT as the head increases in size. In this context, the enrichments of xylem and procambium-specific transcripts (Figure 3) might suggest that aspects of vascular, potentially xylem-like, developmental programs could be a mechanism contributing to stalk elongation and strengthening.

The presence of hormone-responsive pathways (Figure 4 and Figure S3) indicates tight coordination of growth, development, and environmental responses [53]. Plant hormones such as auxin and cytokinin regulate cyclin expression, cell division, and differentiation through complex signalling networks that integrate developmental cues with cell-cycle regulators [54]. Diurnal and developmental regulation of metabolism in CsGTs has been demonstrated, linking hormonal signalling with metabolic activity across trichome tissues [14,15]. In contrast, head tissues were specifically enriched for ethylene signal transduction (Figure S3), consistent with the established role of ethylene as a central regulator of secondary metabolite accumulation in *Cannabis* [55]. Thus, by maintaining the trichomes’ responsiveness to internal and environmental signals, the stalk may act as a primary mediator of systemic and local developmental cues within the trichome, allowing the head to specialize in metabolite production without the constraints of active regulatory mechanisms.

### 3.4. Transport Machinery Is Segregated Between CsGT Tissues

The heatmaps generated for several functional groups, including secondary metabolite production, cell division and growth, transporters and sugar and hormone signalling (Figure 5) showed large numbers of genes consistently differently expressed between head and stalk, further emphasising the contrasting nature of these two CsGT tissues.

Plasmodesmata-associated transcripts, involved in the regulation of plasmodesmata (PD) structure, permeability, and intercellular transport [56,57], showed predominantly higher expression in the stalk, with fewer in the head (Figure 5e). PD are membrane-lined channels enabling selective exchange of metabolites, signals and regulatory molecules, with permeability regulated by callose and PD-associated proteins in response to developmental and environmental cues [58]. In the stalk, Multiple C2-domain and Transmembrane Region Proteins (MCTPs), Plasmodesmata Located Proteins (PDLPs), and callose synthases (CALSs) showed higher expression. PDLPs regulate PD aperture via modulation of callose deposition, CALSs directly mediate callose synthesis, and MCTPs are associated with structurally specialized PD supporting enhanced intercellular connectivity [56,57], collectively supporting a highly regulated and dynamically controllable symplastic connectivity in the stalk. In contrast, the limited expression of PD-related transcripts in the head suggests a more limited modulation of PD permeability focused on concentrating metabolites and limiting backflow.

The upregulation of distinct ABC transporters, aquaporins, cationic amino acid transporters (CATs), amino acid vacuolar transporters (AVTs) and NRT1/PTR (NPF) peptide transporters in the stalk (Figure 5c,d, Tables S9 and S10) indicates enhanced capacity for metabolite transport and sequestration [59,60,61,62,63], water flux and turgor maintenance [64], and amino acid and peptide transport [65,66,67]. Together with elevated ribosomal activity, this supports the stalk as a metabolically active, growth-oriented tissue supplying energy and substrates to the head. Notably, some NPF transporters mediate hormone transport [68], aligning with enriched hormone signaling pathways (Figure 4 and Figure S2), suggesting coordinated regulation of nitrogen availability and developmental cues.

### 3.5. Sugar Signalling and Source-Sink Dynamics in the CsGT

*Hexokinase 1* (*HXK1*), showing higher expression in the head, acts as a sugar sensor regulating allocation and metabolism [69,70,71] while *SNF1-related protein kinase* 1 (*SnRK1*), a key source-sink regulator under energy-limited conditions [72], also showed higher expression in the head, reflecting high energetic demand from cannabinoid and terpenoid synthesis. In a previous study, the SnRK1 protein was more abundant in late-stage *Cannabis* flowers compared to CsGT heads and stalks [13], suggesting post-transcriptional regulation of *SnRK1*. *T6P* signalling via TPS and TPP, which were detected in both CsGT tissues (Figure 5f), regulates the production and breakdown of T6P, a signalling metabolite that reflects cellular sucrose status and helps coordinate nutrient allocation between source and sink tissues [73,74] via inhibition of SnRK1 [75], further demonstrating regulation of SnRK1 and depicting a possible mechanism for coordinating carbohydrate status in CsGTs.

Studies in *Mentha*, *Artemisia*, and *Nicotiana* indicate that secretory cells of non-photosynthetic trichomes primarily receive raffinose-family oligosaccharides (RFOs) as carbon sources [26,76]. CsGTs are likely able to utilise RFOs, as enzymes involved in raffinose synthesis showed higher expression in the head, while α-galactosidases and Galactinol Synthase 1, which mobilize RFOs into simpler sugars, showed higher expression in the stalk [77,78]. Interestingly, α-galactosidase 1 protein accumulated more in the head than in the stalk, suggesting high raffinose utilization in the head (Figure 5f). However, sucrose synthases and invertases [79] showed higher expression in the head, while various sucrose transporters and SWEETs exhibited high expression in both the stalk and head, suggesting that sucrose is also metabolised in CsGTs. The presence of an invertase inhibitor (Figure 5f) in the head indicates tight regulation of sucrose cleavage, enabling fine-tuning of sugar utilization [80].

### 3.6. New Model for CsGT Functioning

**Figure 6:**
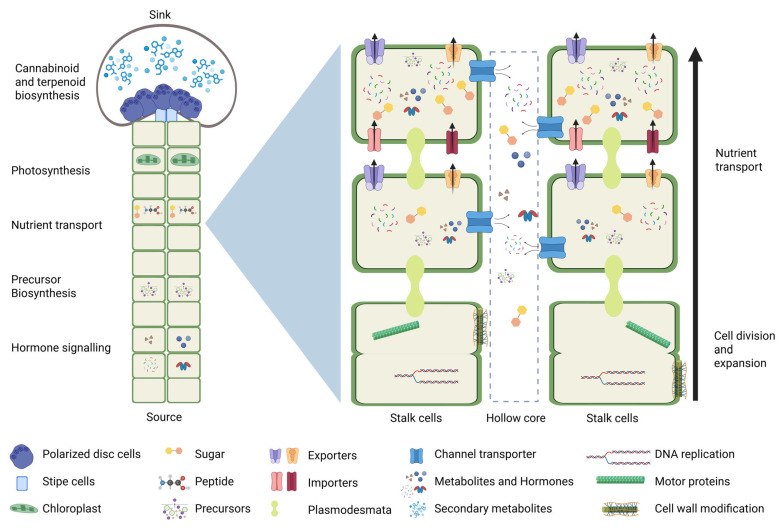
Integrated model of CsGT head and stalk. The head represents a highly specialized biosynthetic compartment and the stalk is a metabolically active tissue, enriched in intercellular transporters and plasmodesmata. Stalk cells undergo longitudinal divisions, contributing to stalk elongation. Evidence also suggests a potential metabolic contribution from the stalk to precursor supply for secondary metabolite biosynthesis and a hollow core for efficient, high-volume sym- and apoplastic transport towards the head. This schematic representation does not imply spatial specialization within the talk, but rather summarizes the functional categories enriched in the stalk transcriptome. Arrow shows nutrient transport directed towards the trichome head. Exporters: ABC, DTX, SWEET transporters; importers: NPF, AVT, CAT, Aquaporins. “Cannabinoid and terpenoid biosynthesis” and “Precursor biosynthesis” refers to data in Figure 5a, “Photosynthesis” refers to data in Figure 4, “Nutrient transport” refers to data in Figure 5c, “Hormone signalling” refers to Figure 4 and Figure S3, “Exporters”, “Importers” and “Channel transporters” refers to data in Figure 5c,d, “Plasmodesmata” refers to data in Figure 5e, “Cell division and expansion” refers to data in Figure 5b, and “Hollow core” refers to a published structural study [5]. Created in BioRender. Siazon, P.M. (2026) (https://BioRender.com/z0hy969, accessed on 21 April 2026).

The transcriptome revealed previously unrecognised functional complexity in the stalk not captured by the proteome. Expression patterns of cell-type-specific marker genes indicated functional complexity and roles related to vascular cell types, while tissue-specific expression patterns for plasmodesmata-associated proteins, aquaporins, SWEETs, and ABC transporters further indicated a stalk optimized for resource allocation, osmotic and redox homeostasis, and sym- and apoplastic connectivity. Collectively, these observations support a model for the stalk and head of CsGTs that has the stalk emerge as a metabolically and regulatory active structure and a specialised transport interface rather than just a passive structural element (Figure 6). The stalk actively grows and acts as a signalling hub coordinating energy and precursor supply, including through a hollow core, whereas the head operates as a highly specialized secretory compartment. The stalk’s complexity contrasted with the head’s specialization shows a division of labour that optimizes secondary metabolite biosynthesis in CsGTs, identifying the stalk as a key contributor to trichome productivity and a potential target for optimizing cannabinoid yield.

## 4. Materials and Methods

### 4.1. Plant Growth and Cultivation

*Cannabis* cultivation, sampling, storage and processing were performed under the Authority granted to Prof. Bronwyn Barkla of Southern Cross University (SCU), issued by the New South Wales Ministry of Health (Australia), in strict adherence to Sections 23(4)(b) and 41(b) of the NSW Drug Misuse and Trafficking Act 1985. Female *C. sativa* plants of the Hindu Kush cultivar were propagated clonally and grown in a controlled growth chamber (Conviron, Conviron®, Grovedale, Australia) set at 28 °C with a long-day photoperiod of 20 h of light and 4 h of darkness (20L/4D) under ambient relative humidity to facilitate root development. Clones were initially rooted in coco peat propagation cubes treated with Clonex^®^ (Growth Techonology, Taunton, UK) rooting gel (3.0 g/L IAA) and, after 7 days, were transferred into 1 L pots containing a 1:1:1 mixture of vermiculite, perlite, and peat moss, supplemented with 1 g/L dolomite and 90 g of Osmocote All Purpose Controlled Release Fertilizer (ICL Growing Solutions, Bella Vista, Australia) (6.4 g/L). Plants were hand-watered daily with tap water. Biological control agents, sourced from Bugs for Bugs (Toowomba, Australia), were applied monthly.

The plants were grown indoors within a growth tent under LED lighting (ViparSpectra© LED, model: R900 W, Richmond, VA, USA) set to conditions that promoted vegetative growth for eight weeks, with an 18:6 h light-dark cycle, and both veg and bloom settings activated. To induce flowering, the light cycle was adjusted to 12 h of light and 12 h of darkness (12L/12D), and the plants were relocated to benches within the grow room. Harvesting was carried out at week 6 of the flowering period.

### 4.2. Isolation of Glandular Trichome Heads and Stalks

Floral tissue from the Hindu Kush cultivar was harvested at week 6 of the flowering period, corresponding to the peak secretory phase of glandular trichome development [2], and trimmed into small floral clusters (*n* = 4 biological replicates, with each replicate derived from an individual plant). One hundred and twenty grams of fresh flower material was submerged in ice-cold ethanol for 15 s using a salad spinner (model # DES0437RD, Isalbi, Brookvale, Australia), followed by draining of the ethanol. The floral clusters were then submerged in a bucket of ice-cold water for 30 s, a process repeated with fresh ice-cold water. The clusters were placed inside a 220 µm mesh bag and transferred to a small washing machine (model # XPB20-1208A, Bubblebagdude, Sherrills Ford, NC, USA) containing five litres of ice-cold water and four litres of ice. The floral tissue underwent a 15 min wash cycle, after which the water was passed through nylon mesh bags with decreasing pore sizes (200 µm, 73 µm, 25 µm). Trichome heads were recovered from the 25 µm sieve and trichome stalks were isolated from the 73 µm sieve using an ice-cold spatula and stored in a prechilled 50 mL Falcon tube containing mannitol buffer (0.2 M mannitol, 0.05 M Tris-HCl, 0.02 M sucrose, 0.005 M MgCl_2_, 0.01 M KCl, 0.0005 M K_2_HPO_4_, and 0.001 M EGTA).

The trichome heads and stalks were pelleted by centrifugation at 10,000 rpm for 10 min at 4 °C in a refrigerated benchtop centrifuge (Heraeus™ Megafuge™ # 75007210, Thermo Fisher Scientific, Allentown, PA, USA), and the supernatant was removed. The samples were flash-frozen in liquid nitrogen and stored at −80 °C.

### 4.3. Confocal Laser Microscopy and Trichome Tissue Counting

All fluorescent images were captured on an Olympus FV-3000 laser scanning confocal microscope (Olympus, Tokyo, Japan) using λ 405 nm, 488 nm, 561 nm, and 640 nm, all at 3% power. Images were all collected in VBF mode, with no line averaging and sequential image capture per line, using a pinhole size of 136 µm. Images for trichome counts were collected in a single imaging plane (XY) at 4096 × 4096 scan size using a UPLSAPO 10×/0.4 NA objective collected using Olympus Fluroview software (FV31S-SW v2.5.1.228) and images were analysed using Olympus cellSens Dimension Desktop software (v4.1, build 26283). ImageJ software (v1.54) [81] was used to manually count stalk and head tissue structures.

### 4.4. Protein Isolation and Digestion of Trichome Proteins

Total protein was isolated using approximately 100 mg of glandular trichome heads and stalks, 4 replicates (*n* = 4 biological replicates, with each replicate derived from an individual plant). Frozen samples were ground to a powder in liquid nitrogen using a mortar and pestle and transferred into pre-chilled 2 mL microcentrifuge tubes. Proteins were precipitated by sequential addition of buffer containing 10× TE, 0.3% (*w*/*v*) sodium deoxycholate, and 72% (*w*/*v*) trichloroacetic acid, with vortexing after each addition. Samples were incubated on ice for 1 h, and the supernatant was discarded by aspiration after centrifugation in a Sigma 4k15 laboratory centrifuge (Sigma Laborzentrifugen GmbH, Osterode am Harz, Germany) at 5525 rcf for 20 min at 4 °C. Pellets were resuspended with ice-cold 90% methanol and then incubated overnight at −20 °C. Pellets were centrifuged at 5525 rcf for 20 min at 4 °C and the supernatant was removed. The pellet was then resuspended with ice-cold 90% methanol and then centrifuged at 5525 rcf for 20 min at 4 °C and the supernatant was removed. This was repeated three times. Protein pellets were then dried in a fume hood and stored at −80 °C until further analysis.

Samples were resuspended in 2 M urea in 50 mM ammonium bicarbonate (pH 8.0) to a final concentration of 1 mg/mL. Proteins were digested with trypsin at an approximate enzyme-to-protein ratio of 1:50 and incubated overnight at 37 °C, following sonication at 50/60 Hz for 10 min (SONICLEAN, Soniclean^®^, Dudley Park, Australia). Samples were subsequently heated briefly (microwave, lowest setting for 4 min), dried using a vacuum centrifuge at 45 °C for 2 h. When dry, samples were dissolved in 100 µL of 1% trifluoroacetic acid in milliQ water, and a 50 µL aliquot was transferred to an autosampler vial for nanoLC-MS/MS system injection.

### 4.5. NanoLC-MS/MS and SWATH Acquisition

LC-MS/MS was performed as previously described [2],. Briefly, peptides were separated on a ChromXP C18CL trap column (3 µm, 120 Å, 350 µm × 0.5 mm) followed by an analytical column (3 µm, 120 Å, 75 µm × 15 cm). A 90 min linear gradient was applied, increasing from 2% to 35% acetonitrile in 0.1% formic acid at a flow rate of 300 nL/min. The SWATH-MS acquisition scheme consisted of a TOF MS1 survey scan (*m*/*z* 350–1500, 100 ms accumulation) followed by 100 variable-width MS2 windows covering *m*/*z* 350–1500 (40 ms accumulation per window), resulting in a cycle time of ~4.2 s. Ion source parameters included an ion spray voltage of 2.5 kV and an interface heater temperature of 150 °C. The instrument was calibrated daily using the β-galactosidase reference standard to ensure mass accuracy.

### 4.6. Protein Identification and Quantification

Protein identification and quantification were performed using DIA-NN (v.1.8.1) in library-free mode. A two-pass strategy was applied: a deep learning-predicted spectral library was first generated directly from the DIA runs using in silico digestion of a *C. sativa* protein FASTA database (GCF_029168945.1_ASM2916894v1.protein.faa) [82], followed by reanalysis using the refined library. Digestion was performed with trypsin specificity, allowing up to one missed cleavage, and filtering peptides to a length range of 7–30 amino acids.

Precursor and fragment ion *m*/*z* ranges were set to 300–1800 and 200–1800, respectively. DIA-NN automatically optimized MS1 and MS2 mass accuracy parameters during the first pass. Quantification was based on fixed-width peak apex intensities, and interference removal was disabled. Normalization across runs was performed using the built-in Most Likely Ratio (MLR) method to correct for systematic variation. The false discovery rate (FDR) was controlled at 1% at both precursor and protein group levels using proteotypic peptides only. Protein inference was performed at the protein group level using highly heuristic grouping to minimize redundancy.

### 4.7. RNA Extraction, Sequencing, and Mapping

Total RNA was isolated from the same biological material used for protein extraction, with approximately 100 mg of glandular trichome heads and stalks, 4 replicates (*n* = 4 biological replicates, with each replicate derived from an individual plant). Frozen samples were homogenised using a Qiagen Tissuelyzer II (Qiagen, Hilden, Germany) at 1500 rpm for 30 s in a 2 mL microcentrifuge tube containing 2× 4.5 mm stainless steel grinding balls under cryogenic conditions. Total RNA was isolated using the Qiagen RNeasy Plant Mini Kit (Qiagen, Hilden, Germany) with buffer RLC and DNase I on-column digestion performed according to the manufacturer’s instructions.

Quantification and quality check of extracted RNA were done using Agilent 2100 Bioanalyzer RNA 6000 Kits (Agilent Technologies, Santa Clara, CA, USA). Samples were shipped to the Australian Genome Research Facility Ltd. (AGRF, Melbourne, Victoria, Australia) for mRNA library preparation and sequencing. Sequencing was performed on an Illumina NovaSeq platform using 150 bp paired-end reads.

Downloaded reads in FASTQ format were processed using CLC Genomics Workbench 11.0 (QIAGEN, Germantown, MD, USA). Reads were mapped against the *C. sativa* Pink Pepper genome [82] and transcript quantification (TPM, Transcripts Per Million) was performed in CLC Genomics Workbench 11.0.

### 4.8. Cell-Type Marker Analysis

#### 4.8.1. Selection of Arabidopsis Cell-Type Marker Genes

Arabidopsis cell-type marker genes were obtained from a leaf single-cell RNA-seq study that characterized transcriptional signatures of distinct leaf cell populations [21]. Genes were selected based on their consistent enrichment within defined cell clusters. The final set included markers representing major leaf cell types: phloem parenchyma (*SWEET12*), companion cells (*SUC2*, *FTIP1*, *APL*), bundle sheath (*SCL23*, *AST56*), xylem (*GLR3.6*, *ACL5*), mesophyll (*LHCB2.1*, *CA1*), spongy mesophyll (*AFO*), procambium (*MP*, *PXY*, *WOX4*), epidermis (*DUF538*, *ATML1*, *KCS6*), guard cells (*FMA*, *MPK9*), and hydathodes (*EP3*).

#### 4.8.2. Identification of *Cannabis sativa* Homologues by Reciprocal Best-Hit Analysis

Protein sequences corresponding to Arabidopsis marker genes (Araport11) [83] were queried against the *C. sativa* (Pink pepper) reference proteome using DIAMOND (v2.1.16.) in BLASTP mode with default parameters. The top-scoring *C. sativa* hits meeting minimum thresholds (E-value ≤ 1 × 10^−10^; query coverage ≥ 50%) were then reciprocally queried against the Arabidopsis Araport11 proteome using the same approach and settings.

Gene pairs were retained as homologues only when each represented the best hit of the other in the reciprocal searches. Only marker genes with RBH-supported *C. sativa* homologues were retained for downstream expression analyses.

#### 4.8.3. Bulk RNA-Seq Expression Quantification of Cell-Type Marker Genes in Trichome Tissues

Transcript abundance of *C. sativa* cell-type marker homologues was quantified using TPM-normalized bulk RNA-seq data generated from isolated trichome head and trichome stalk tissues. TPM-normalized abundance values for leaf tissue were obtained from the publicly available Cannabis Expression Atlas [22] and used as a reference tissue for comparative interpretation of expression patterns.

### 4.9. Bioinformatic and Statistical Analyses

All statistical analyses and plots were generated in R (v4.4.0) [84] through RStudio (v2026.1.1.403, Apple Blossom) [85] using R/Tidyverse (v2.0.0) [86], R/DESeq2 (v1.44.0) [87], R/Pathview (v1.44.0) [88], and R/patchwork (v.1.3.2) [89] packages. Principal component analysis (PCA) was conducted to evaluate sample clustering and to assess variance attributable to tissue type before proceeding with differential expression analysis.

Raw count data were processed using DESeq2 (v1.44.0) to identify differentially expressed genes (DEGs) between trichome head and stalk tissues. Additional visualizations, including volcano plots and hierarchical clustering heatmaps, were generated in R to examine DEG expression patterns.

Functional annotations were retrieved directly from the KEGG organism-specific database entry for Pink Pepper (T08987), with pre-assigned GO/KEGG functional mappings for the corresponding gene identifiers.

## Data Availability

The raw mass spectrometry proteomics data and RNA-seq data generated in this study have been deposited to the Southern Cross University research portal (https://researchportal.scu.edu.au/) (accessed on 18 April 2026)) and are available under DOI https://doi.org/10.26918/data.562 (accessed on 18 April 2026).

## Supplementary Materials

The following supporting information can be downloaded at: https://www.mdpi.com/article/10.3390/plants15111624/s1, Figure S1: Microscopy counts of tissue purity in isolated CsGT head and stalk fractions; Figure S2: Principal component analysis of proteomic and transcriptomic datasets from CsGT stalk and head tissues; Figure S3: Plant hormone signal transduction pathway map showing differential transcript abundance between CsGT stalk and head tissues Transcripts; Figure S4: GO Biological Process (BP) enrichment analysis of CsGT stalk and head tissues; Figure S5: Expression of phloem-associated transcription factors and vascular development genes in CsGT stalk and head tissues; Table S1: Summary of transcriptome sequencing data; Table S2: Summary of Stalk vs. Head DAP analysis; Table S3: Summary of Stalk vs. Head DEG analysis; Table S4: Summary of concordance data between DAP and DEG; Table S5: Top 20 most abundant proteins and transcripts; Table S6: Expression data of cell type marker genes used for cell identity annotation; Table S7: Annotations and normalised count z-scores of secondary metabolism related transcripts; Table S8: Annotations and normalised count z-scores of cell growth related transcripts; Table S9: Annotations and normalised count z-scores of amino acid, SWEET, and peptide transporter related transcripts; Table S10: Annotations and normalised count z-scores of ABC, DTX, and Aquaporin related transcripts; Table S11: Annotations and normalised count z-scores of Plasmodesmata related transcripts; Table S12: Annotations and normalised count z-scores of source-sink related transcripts.

## Abbreviations

The following abbreviations are used in this manuscript:

CsGT	*Cannabis sativa* glandular trichome
THCA	Δ9-tetrahydrocannabinolic acid
CBDA	Cannabidiolic acid
MEP	methylerythritol phosphate
ATP	Adenosine triphosphate
RNA	Ribonucleic acid
IDA	Information-dependent acquisition
UV	Ultraviolet
PCA	Principal component analysis
PC1	Principal component 1
SWATH-MS	Sequential window acquisition of all theoretical mass spectra
DAPs	Differentially abundant proteins
DEGs	Differentially expressed genes
mRNA	messenger RNA
ER	Endoplasmic reticulum
*DUF538*	Domain of unknown function 538
*KCS6*	Ketoacyl-Coenzyme A synthase 6
*ATML1*	Arabidopsis thaliana meristem layer 1
*SWEET12*	Sugars will eventually be exported transporter 12
*SUC2*	Sucrose transport protein 2
*FTIP1*	Flowering locus T interacting protein 1
*APL*	Altered phloem development
*SCL23*	Scarecrow-like 23
*AST56*	Arabidopsis sulfate transporter 56
*GLR3.6*	Glutamate receptor-like 3.6
*ACL5*	Acaulis 5
*LHCB2.1*	Light-harvesting chlorophyll a/b-binding protein 2.1
*CA1*	Carbonic anhydrase 1
*AFO*	Abnormal floral organs
*MP*	Monopteros
*PXY*	Phloem intercalated with xylem
*WOX4*	Wuschel-related homeobox 4
*FMA*	Fama
*MPK9*	Mitogen-activated protein kinase 9
*EP3*	Endochitinase EP3
KEGG	Kyoto Encyclopedia of genes and genomes
DNA	Deoxyribonucleic acid
ABC	ATP-binding cassette
TCA	Tricarboxylic acid
GO	Gene ontology
BP	Biological process
AOS	Allene oxide synthases
THCAS	Tetrahydrocannabinolic acid synthase
CBCAS	Cannabichromenic acid synthase
CBDAS	Cannabidiolic acid synthase
OAC	Olivetolic acid cyclase
APT	2-acylphloroglucinol 4-prenyltransferase
TPS1	Limonene synthase 21
MYRS	Myrcene synthase
LOX	Lipoxygenase
CYC	Cyclin
CDK	Cyclin-dependent kinase
CKI	CDK inhibitor
EXPA	α-expansin
EXPB	β-expansin
SMR	Siamese-related
AVT	Amino acid vacuolar transporter
CAT	Cationic amino acid transporter
NPF	Nitrate transporter1/Peptide transporter family transporters
MATE	Multidrug and toxic compound extrusion transporters
DTX	Detoxification
PIP	Plasma membrane intrinsic protein
TIP	Tonoplast intrinsic protein
PD	Plasmodesmata
PDLP	Plasmodesmata-located proteins
PDCB	Plasmodesmata callose-binding proteins
CALS	Callose synthase
REM	Remorin
MCTP	Multiple C2 domains and transmembrane region proteins
SUT1	Sucrose transport protein 1
SUS7	Sucrose synthase 7
AGAL	α-Galactosidase
TPS	Trehalose phosphate synthases
TPP	Trehalose phosphate phosphatases
GOLS	Galactinol synthase
AINV	Alkaline/Neutral Invertase
nsLTP1	Non-specific lipid transfer protein 1
scRNA	Single-cell RNA
HXK1	Hexokinase 1
SNF1	Sucrose non-fermenting 1
SnRK1	SNF1-related protein kinase 1
T6P	Trehalose 6-phosphate
RFO	Raffinose-family oligosaccharide
IAA	Indole-3-acetic acid
LED	Light-emitting diode
Tris-HCl	Tris hydrochloride
MgCl_2_	Magnesium chloride
KCl	Potassium chloride
K_2_HPO_4_	Dipotassium phosphate
EGTA	Ethylene glycol-bis(β-aminoethyl ether)-N,N,N’,N’-tetraacetic acid
EDTA	Ethylenediaminetetraacetic acid
TE	Tris-EDTA
LC-MS/MS	Liquid chromatography-tandem mass spectrometry
DIA-NN	Data-independent acquisition by neural networks
MLR	Most likely ratio
FDR	False discovery rate
