# Supplementary material for: Heads Up: Transcriptomics Reveal Functional Roles of Cannabis Glandular Trichome Stalks
Source: Plants (Basel). 2026 May 26;15(11):1624. doi: 10.3390/plants15111624 (PMC13259226; doi:10.3390/plants15111624)
Supplement: Supplementary file 1 [file plants-15-01624-s001.zip › Supplementary figures v2.pdf]

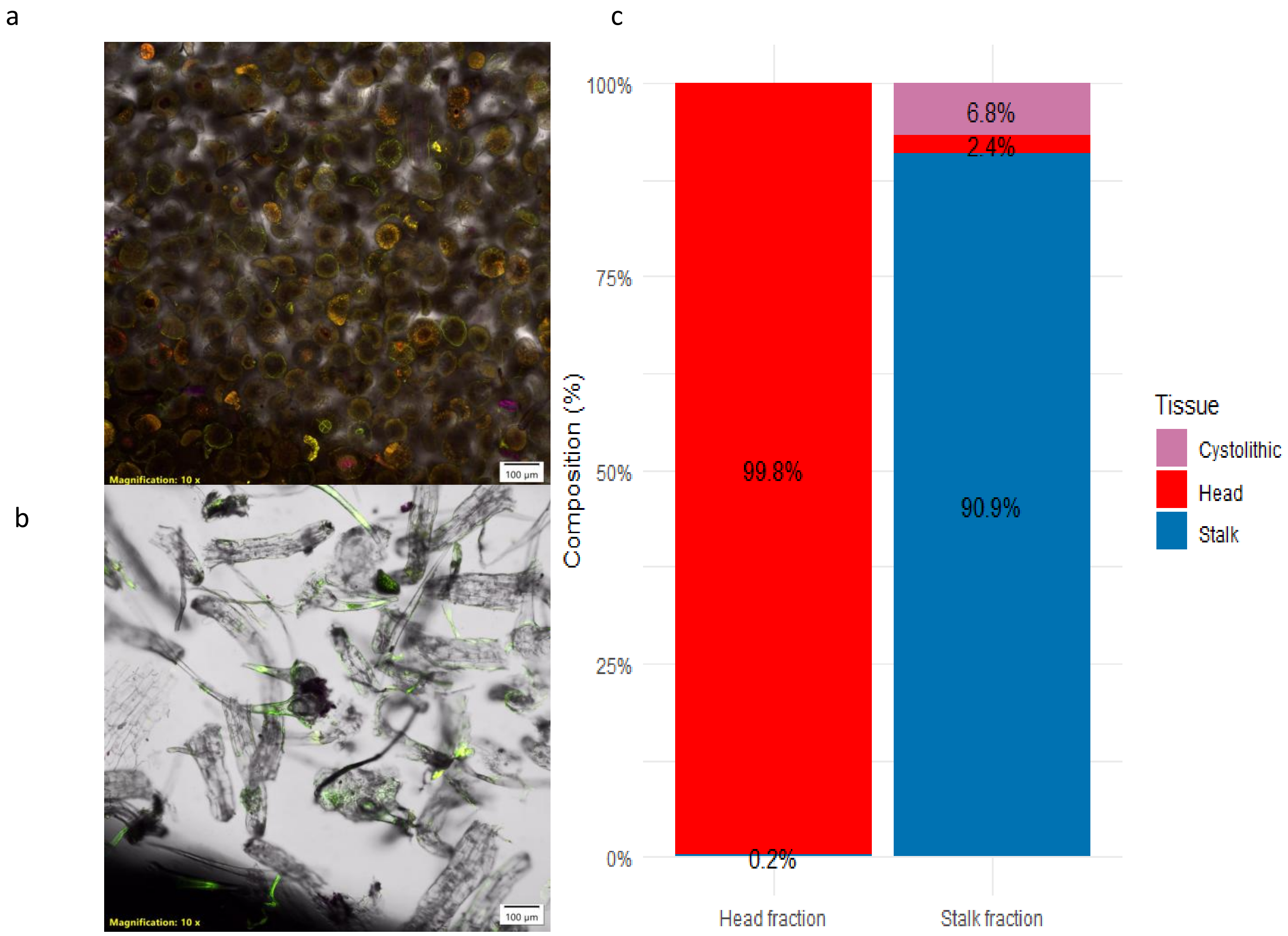

Figure S1. Microscopy counts of tissue purity in isolated CsGT head and stalk fractions. (a) Representative microscopy image of the isolated head fraction showing predominantly heads. (b) Representative microscopy image of the isolated stalk fraction showing stalk structures. (c) Quantification of tissue purity based on manual counting of structures expressed as the percentage of tissue types in each fraction. The head fraction contained 99.8% head tissue, while the stalk fraction contained 90.9% stalk tissues.

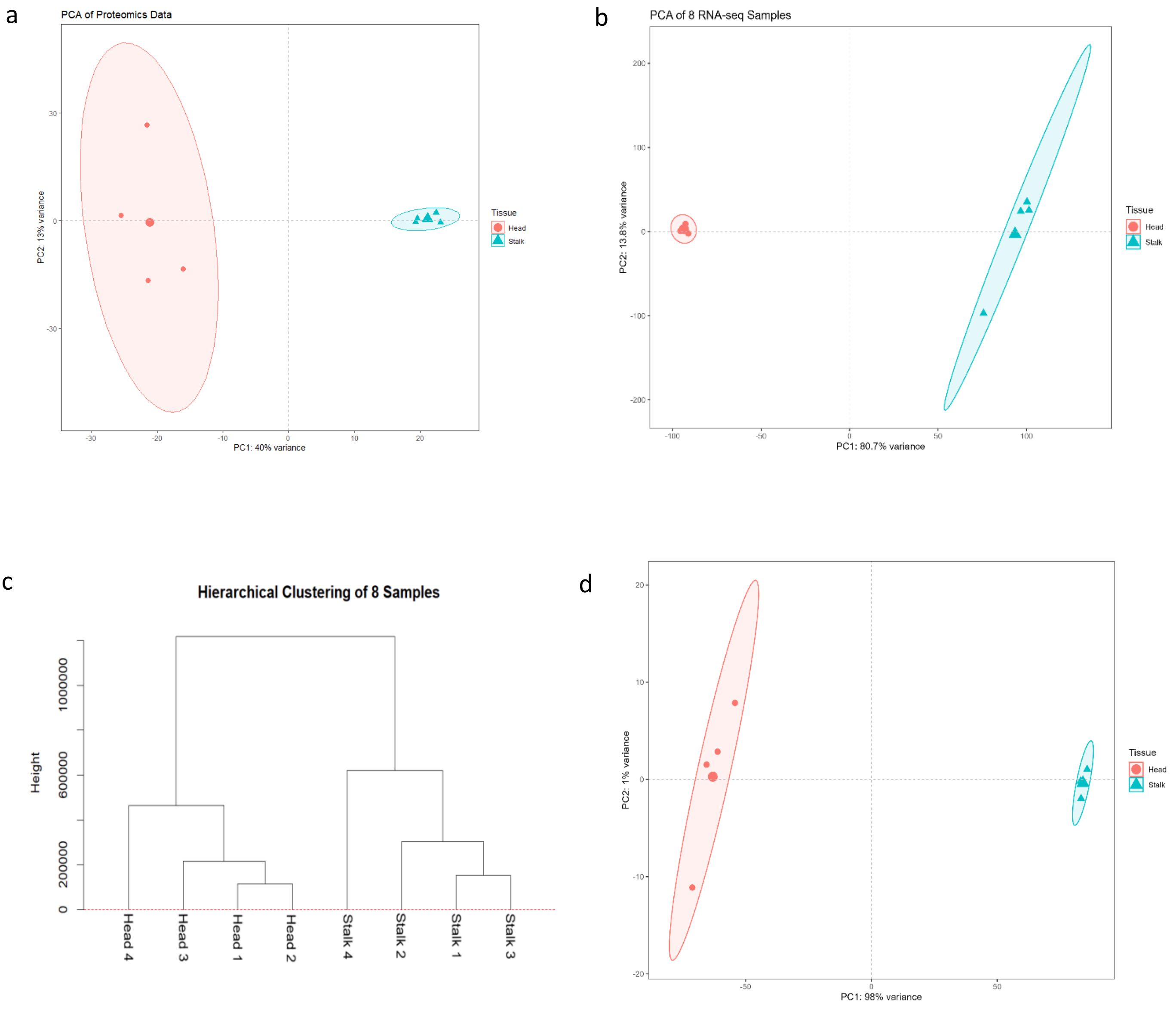

Figure S2. Principal component analysis (PCA) of proteomic and transcriptomic datasets from CsGT stalk and head tissues. (a) PCA of the proteomic dataset showing clear separation between head and stalk samples along PC1 with 40.0% variance explained. (b) PCA of the transcriptomic dataset showing strong separation between tissues along PC1 with 80.7% variance explained, with one outlier observed in the stalk samples. (c) Hierarchical clustering of RNA-seq samples (n = 8) confirming tissue-specific grouping, while also indicating that stalk sample 4 is the least related to the other stalk replicates. (d) PCA of the transcriptomic dataset after removal of sample Stalk 4, resulting in improved clustering and increased separation between tissues, with PC1 explaining 98.0% of the variance.

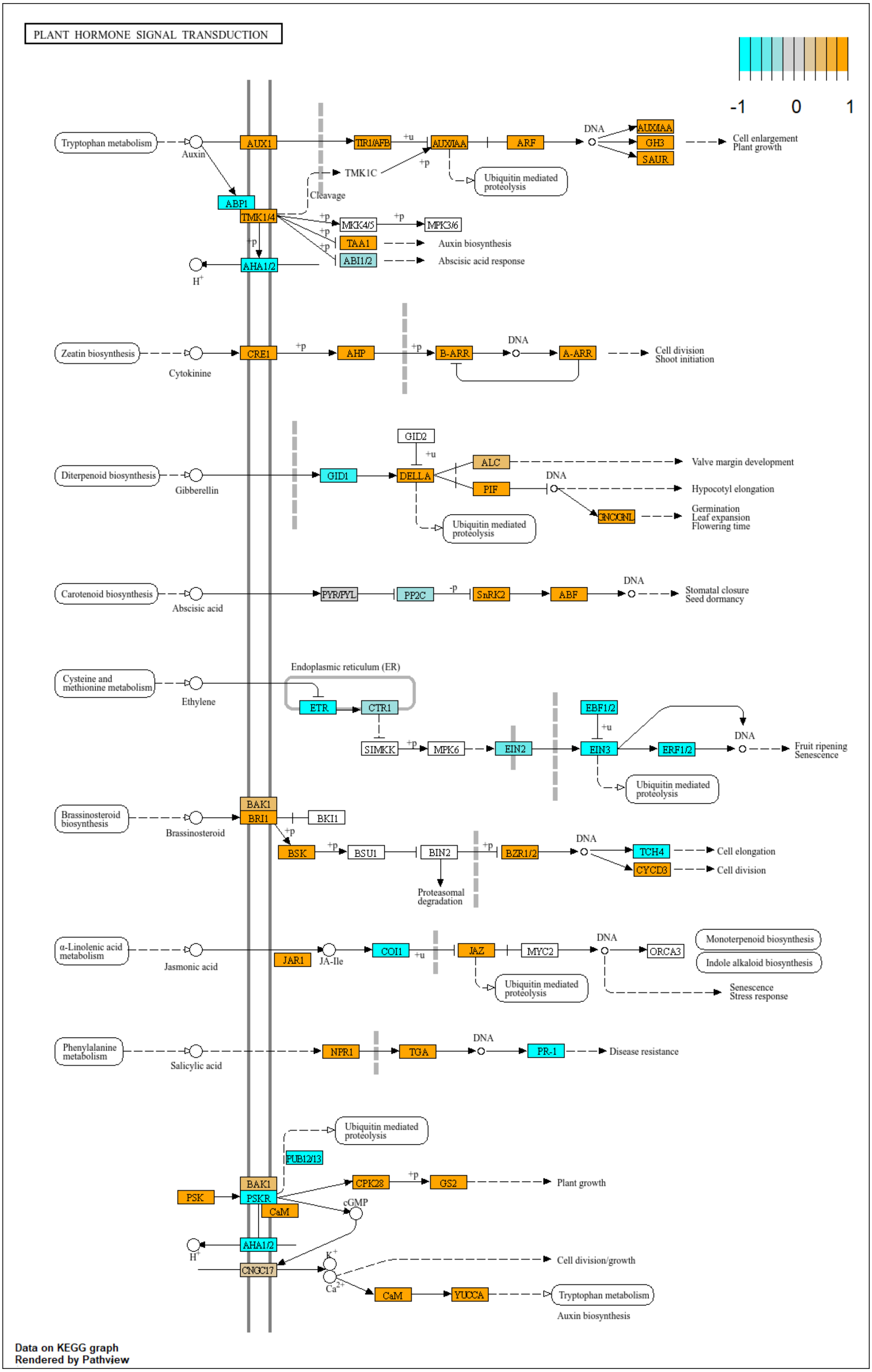
PLANT HORMONE SIGNAL TRANSDUCTION
-1
0
1
Tryptophan metabolism
Auxin
AUX1
TIR1/AFB
+u
AUX/IAA
ARF
DNA
AUX/IAA
GH3
SAUR
Cell enlargement
Plant growth
+p
TMK1C
Cleavage
Ubiquitin mediated proteolysis
ABP1
TMK1/4
+p
MKK4/5
MPK3/6
TAA1
Auxin biosynthesis
ABI1/2
Abscisic acid response
AHA1/2
H+
Zeatin biosynthesis
Cytokinine
CRE1
AHP
B-ARR
A-ARR
Cell division
Shoot initiation
Diterpenoid biosynthesis
Gibberellin
GID1
GID2
DELLA
ALC
Valve margin development
PIF
Hypocotyl elongation
GNC/GNL
Germination
Leaf expansion
Flowering time
Carotenoid biosynthesis
Abscisic acid
PYR/PYL
PP2C
-p
SnRK2
ABF
Stomatal closure
Seed dormancy
Cysteine and methionine metabolism
Ethylene
Endoplasmic reticulum (ER)
ETR
CTR1
SIMKK
MPK6
EIN2
EBF1/2
EIN3
ERF1/2
Fruit ripening
Senescence
Brassinosteroid biosynthesis
Brassinosteroid
BAK1
BRI1
BKI1
BSK
BSU1
BIN2
Proteasomal degradation
BZR1/2
TCH4
CYCD3
Cell elongation
Cell division
α-Linolenic acid metabolism
Jasmonic acid
JAR1
JA-Ile
COI1
JAZ
MYC2
ORCA3
Monoterpenoid biosynthesis
Indole alkaloid biosynthesis
Senescence
Stress response
Phenylalanine metabolism
Salicylic acid
NPR1
TGA
PR-1
Disease resistance
PUB12/13
PSK
PSKR
CaM
CPK28
GS2
Plant growth
cGMP
K+
Ca2+
CNGC17
Cell division/growth
YUCCA
Tryptophan metabolism
Auxin biosynthesis
Data on KEGG graph
Rendered by Pathview

Figure S3. Plant hormone signal transduction pathway map showing differential transcript abundance between CsGT stalk and head tissues. Transcripts highlighted in orange indicate higher expression in CsGT stalk, whereas transcripts highlighted in blue indicate higher expression in CsGT head. The pathway was generated using Pathview R package. Abbreviations: AUX1 – Auxin influx carrier 1; TIR1/AFB – Transport inhibitor response 1 / Auxin signaling F-box proteins; AUX/IAA – Auxin/Indole-3-acetic acid proteins; ARF – Auxin response factor; GH3 – Gretchen Hagen 3 (auxin-responsive acyl acid amido synthetase); SAUR – Small auxin up RNA protein; ABP1 – Auxin-binding protein 1; TMK1/4 – Transmembrane kinase 1/4; TMK1C – Transmembrane kinase 1 C-terminal fragment; AHA1/2 – Arabidopsis H^+-ATPase 1/2; MPK3/6 – Mitogen-activated protein kinase 3/6; CRE1 – Cytokinin response 1 (histidine kinase receptor); AHP – Arabidopsis histidine phosphotransfer protein; B-ARR – Type-B Arabidopsis response regulator; A-ARR – Type-A Arabidopsis response regulator; GID1 – Gibberellin insensitive dwarf 1 (gibberellin receptor); DELLA – DELLA protein (gibberellin signaling repressor); GID2 – Gibberellin insensitive dwarf 2 (F-box protein); ALC – Alcatraz transcription factor; PIF – Phytochrome-interacting factor; GNC/GNL – GATA nitrate-inducible carbon-metabolism involved / GNC-like; PYR/PYL – Pyrabactin resistance / PYR1-like abscisic acid receptors; PP2C – Protein phosphatase 2C; SNRK2 – SNF1-related protein kinase 2; ABF – ABA-responsive element-binding factor; ETR – Ethylene receptor; CTR1 – Constitutive triple response 1; SIMKK – Stress-induced MAP kinase kinase; MPK6 – Mitogen-activated protein kinase 6; EIN2 – Ethylene-insensitive protein 2; EIN3 – Ethylene-insensitive protein 3; EBF1/2 – EIN3-binding F-box protein 1/2; ERF1/2 – Ethylene-responsive transcription factor 1/2; BAK1 – BRI1-associated receptor kinase 1; BRI1 – Brassinosteroid insensitive 1 (receptor kinase); BKI1 – BRI1 kinase inhibitor 1; BSK – Brassinosteroid signaling kinase; BSU1 – BRI1 suppressor 1 (protein phosphatase); BIN2 – Brassinosteroid insensitive 2 (GSK3-like kinase); BZR1/2 – Brassinazole-resistant 1/2; TCH4 – Touch 4 (xyloglucan endotransglucosylase); CYCD3 – Cyclin D3; JAR1 – Jasmonate resistant 1 (jasmonic acid–amino acid synthetase); COI1 – Coronatine-insensitive protein 1; JAZ – Jasmonate ZIM-domain protein; MYC2 – Transcription factor MYC2; ORCA3 – Octadecanoid-responsive Catharanthus AP2-domain protein 3; NPR1 – Nonexpressor of pathogenesis-related genes 1; TGA – TGACG motif-binding transcription factor; PR-1 – Pathogenesis-related protein 1; PSK – Phytosulfokine; PSKR – Phytosulfokine receptor; CaM – Calmodulin; PUB12/13 – Plant U-box protein 12/13; CNGC17 – Cyclic nucleotide-gated channel 17; CPK28 – Calcium-dependent protein kinase 28; GS2 – Glutamine synthetase 2; YUCCA – Indole-3-pyruvate monooxygenase (flavin monooxygenase).

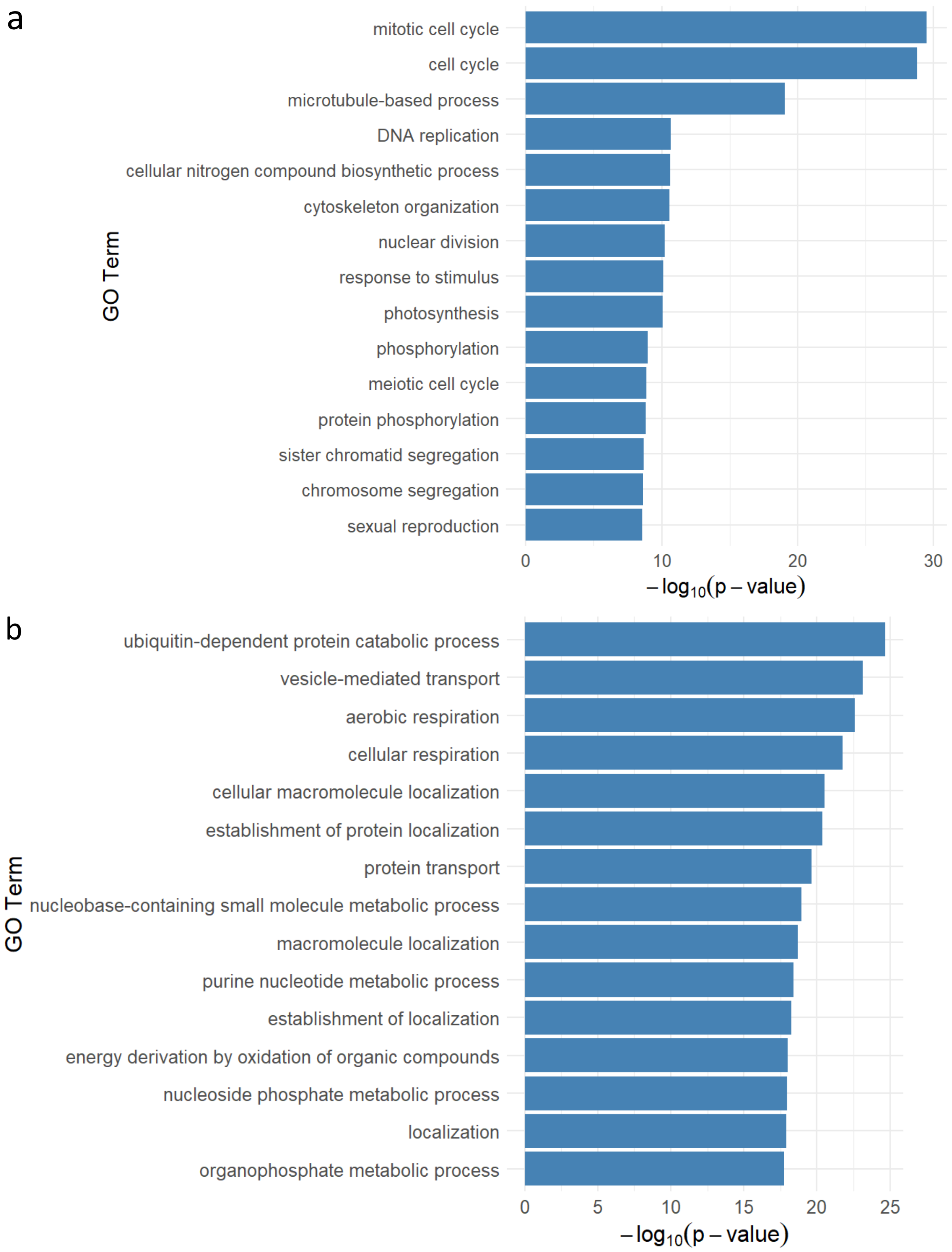

Figure S4. GO Biological Process (BP) enrichment analysis of CsGT stalk and head tissues. (a) GO BP terms enriched in genes upregulated in CsGT stalk tissue. (b) GO BP terms enriched in genes upregulated in CsGT head tissue. Enrichment analysis was performed using Fisher's exact test in topGO. GO terms were filtered using an FDR (padj) threshold of < 0.05 and a minimum gene count of 3–5, and the top 15 most significantly enriched terms (ranked by p-value) were visualised for each tissue.

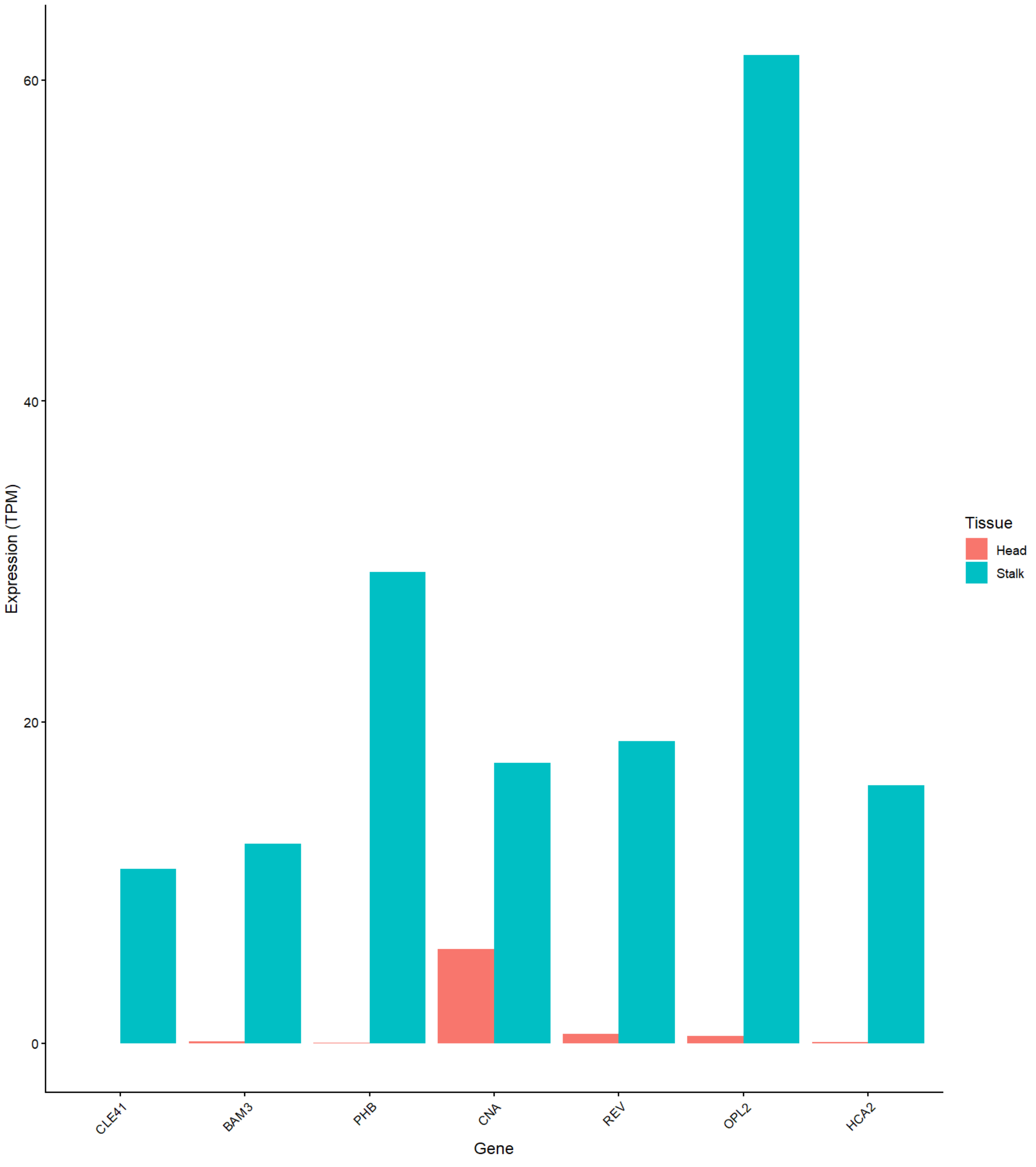

Figure S5. Expression of phloem-associated transcription factors and vascular development genes in CsGT stalk and head tissues. Abbreviations: CLE41 – CLAVATA3/ESR-related protein 41; BAM3 – Barely any meristem 3; PHB – Phabulosa; CNA - Corona; REV - Revoluta; OPL2 – Octopus like 2 ; HCA2 – High cambial activity 2.
